# Inulin may inhibit ADAM17 to delay atherosclerosis

**DOI:** 10.1515/med-2026-1433

**Published:** 2026-05-19

**Authors:** Wang Lijuan, Du Jing, Liang Xuegang

**Affiliations:** Department of Endocrinology, Ningxia Hui Autonomous Region People’s Hospital, Ningxia Medical University, Yinchuan City, Ningxia, China; Cardiovascular Department of the Second People’s Hospital of Yinchuan, Yinchuan City, Ningxia, China; Ningxia Hui Autonomous Region People’s Hospital, Ningxia Medical University, Yinchuan City, Ningxia, China

**Keywords:** inulin, ADAM17, inflammation, immunity, atherosclerosis

## Abstract

**Objectives:**

This study sought to elucidate the mechanism by which inulin delays atherogenesis, specifically investigating its potential to mitigate inflammation through the inhibition of ADAM17.

**Methods:**

A cohort of 180 patients (aged 18–75, mean 55 ± 15.4 years) was recruited from Yinchuan Second People’s Hospital (Jan 2021–Jan 2023). Based on carotid artery examinations, participants were stratified into three groups: healthy, plaque-free dyslipidemia (PFD), and atherosclerotic. The following parameters were analyzed: ① serum ADAM17 levels, ② inflammatory markers, and ③ lipid profiles (triglycerides and low-density lipoprotein). Complementary *in vitro* experiments were conducted to validate the interaction between inulin and ADAM17-mediated inflammatory pathways.

**Results:**

Serum ADAM17 levels exhibited significant stepwise increases across the patient groups, with marked differences between the healthy, PFD, and atherosclerotic groups (p<0.001). Cellular assays confirmed that inulin administration significantly counteracted the upregulation of IL-1β (p<00.001) induced by ADAM17. Furthermore, IL-6 was found to promote ADAM17 expression, an effect that was significantly suppressed by inulin treatment (p<00.001), demonstrating a clear antagonistic relationship.

**Conclusions:**

The findings indicate that inulin attenuates atherosclerosis by suppressing ADAM17 expression and its downstream inflammatory cascade. These results provide a strategic insight and a novel perspective for leveraging inflammation suppression to improve patient prognosis.

## Introduction

The pathogenesis of atherosclerosis (AS) is unclear and is currently considered to be a chronic inflammatory disease of the arterial vessel wall caused by an imbalance in lipid metabolism which is the pathological basis of a number of vascular diseases such as coronary artery disease, cerebral infarction and peripheral vascular disease [1], 2]. In addition, the incidence of various vascular diseases caused by AS is increasing every year, endangering the quality of life of patients [3]. Therefore, it is important to actively search for more effective therapeutic approaches to treat AS and elucidate their therapeutic mechanisms as well as explore sensitive and specific targets at the molecular level that may help in the diagnosis, treatment and prognosis of AS (Table 1).

**Table 1:** Baseline characteristics of the study population.

Characteristics	All patients (n=180)	Healthy group (n=60)	PDF group (n=60)	As group (n=60)	p-Value
Age, years Mean ± SD	55.0 ± 15.4	50.5 ± 10.2	57.1 ± 9.8	58.5 ± 10.2	0.11
Male n (%)	77 (42.8)	27 (45.0)	26 (43.3)	27 (40.0)	0.45
Female n (%)	103 (57.2)	33 (55.0)	34 (56.7)	36 (60.0)	0.08
Diabetes n (%)	40 (22.2)	13 (21.7.0)	13 (21.7.0)	14 (23.3)	0.30
Smoker n (%)	54 (30.0)	19 (31.7)	17 (28.3)	18 (30.0)	0.17

Recent studies have shown that chronic inflammatory reactions also play a notable role in the development of AS [4], 5]. In early stages, AS is caused by endothelial damage and abnormal lipid metabolism, leading to inflammatory changes mediated by vascular endothelial cells (ECs) [6]. When ECs are activated, monocyte chemoattractant protein 1 (MCP-1), interleukin (IL)-1β, IL-6, tumor necrosis factor-α (TNF-α), intercellular adhesion molecule-1 (ICAM-1), vascular cell adhesion molecule-1 (VCAM-1) as well as other inflammatory factors attract lymphocytes and monocytes to bind to the ECs and infiltrate the arterial wall, and inflammation occurs [7], 8]. In the advanced stage of AS, vascular ECs secrete matrix metalloproteinases (MMPs), which degrade collagen fibers in the extracellular plaque matrix, leading to plaque rupture, hemorrhage and thrombosis [9]. The synergistic action of all inflammatory factors in plaques not only increases inflammation, but also prevents the renewal of structural elements which are mechanically stable in inflamed tissues [10], 11]. Therefore, reducing inflammatory factors is a good guide for the anti-AS aspect, slowing down the progression of the disease from multiple aspects, and it is possible that anti-inflammatory treatments have good clinical application prospects for alleviating AS lesions in the future.

In recent years, a deintegrin metalloproteinases (ADAMs), secreted glycoproteins with multiple functions [12], 13], have been shown to play an important role in vascular pathology and physiological processes. ADAM17, a key enzyme in the activation and cleavage of various cytokines, is involved in vascular pathophysiology [14], 15]. Wen et al. and others [16], [17], [18] suggested that the increased activity of ADAM17 could increase the risk of cardiovascular events, suggesting the value of monitoring ADAM17. These studies preliminarily tentatively suggested that ADAM17 may be associated with AS lesions. However, the involvement of ADAM17 in the development of AS, the effects on adhesion molecules and inflammatory factors in AS plaques and the clinical feasibility of small molecule inhibitory analogues targeting ADAM17 still require further investigation.

Inulin (INU), a type of plant-derived fiber, is not digested or absorbed by the human body. As an important prebiotic, it has been demonstrated to ameliorate various chronic metabolic disorders, such as alcoholic liver disease [19], non-alcoholic fatty liver disease [20], polycystic ovary syndrome [21], type 2 diabetes [22], and colitis [23]. Our previous study revealed the protective effects of INU against atherosclerosis (AS) in apolipoprotein E-deficient (ApoE^−/−^) mice, which are prone to AS [24]. Furthermore, INU has been shown to improve metabolic function and regulate intestinal immunity [25]. In clinical practice, we observed that patients with chronic inflammation are at a higher risk of developing atherosclerosis. The purpose of this study was to investigate whether ADAM17, as a key inflammatory mediator, is involved in this process, and whether inulin alleviates vascular inflammation by inhibiting ADAM17.

## Materials and methods

### Human serologic study

The study subjects were 180 patients who visited the Cardiovascular Department of the Second People’s Hospital of Yinchuan, Ningxia from January 2021 to January 2023. There were 80 males and 100 females, aged 18–75 years, average age, 55 ± 15.4 years. 43 patients had hypertension (defined as blood pressure ≥140/90 mmHg or being on antihypertensive medication) and 20 patients had type 2 diabetes (defined as fasting blood glucose ≥7.0 mmol/L or HbA1c ≥6.5 %). According to the results of color Doppler ultrasound and blood lipid examination of carotid artery, they were divided into healthy group (both normal), Plaque-free dyslipidemia group (PFD) (with hyperlipidemia but no plaque on color Doppler ultrasound of carotid artery) and atherosclerosis group (with plaque and hyperlipidemia on color Doppler ultrasound), with 60 cases in each group. Inclusion criteria: ① No metastasis; ② No history of radiotherapy or chemotherapy; ③ No other serious cardiovascular diseases, malignant tumors or other comorbidities; ④ No history of long-term use of antibiotics; ⑤ Those who voluntarily participate in this study and sign the informed consent form. Exclusion criteria: ① Pregnant or lactating women; ② Individuals with abnormal thyroid or adrenal function; ③ Individuals with severe liver and kidney dysfunction, immune system diseases, and other comorbidities; ④ Participants in other clinical trials. The observation indicators were the following: ① Serum levels of ADAM17; ② serum levels of inflammatory markers, such as C-reactive protein (CRP), erythrocyte sedimentation rate (ESR), IL-6 and others; and ③ triglyceride (TG) and low-density lipoprotein (LDL) levels. This study was approved by the Ethics Committee of the Second People’s Hospital of Yinchuan, Ningxia (approval no. NZR-003).

### Cytologic level studies

*Culture of human umbilical vein ECs (HUVECs, ATCC, USA)*. Human Umbilical Vein Endothelial Cells (HUVEC) were used for the test, and the test steps were as follows: ① take out the frozen cells for resuscitation ② change the cytosol ③ change the medium every 2 days ④ cultivate for 5–7 days ⑤ after the cells climbed up to 80 %, add ADAM17(Concentration 0.1 ng/L), Inulin (Concentration gradient: 0.1, 1, 10 mg/mL, exposure time: 24, 48, 72 h dissolved in serum-free medium), and IL-6(Concentration 0.1 pg/mL), respectively, and divide into two parts: one part was divided into the control group, ADAM17+Inulin group; the other part was divided into the control group, IL-6 group, and Inulin group, and cultured again for 10 days. After the cells reached 80 %, ADAM17, Inulin and IL-6 were added respectively, and the cells were divided into two parts: one part was divided into the control group, ADAM17 group and ADAM17+Inulin group; the other part was divided into the control group, IL-6 group and Inulin group, and then cultured for 10 days, and then RNA and protein were extracted, and then RNA was detected by the machine, and then Western blot experiments were carried out in order.

*Reverse transcription-quantitative PCR (RT-qPCR)*. Total RNA (RNA extraction using TRIzol method) was isolated from cell tissues using a high-speed homogenizer reagent, followed by RT using the isolated RNA (1 μg) with a PrimeScript RT kit. RT-qPCR analysis was performed using LightCycler 480 SYBR-Green I Master.

*Western blotting (Internal reference gene: GAPDH)*. Adherent cells were collected, lysis buffer was added to extract proteins, colloids were prepared, and electrophoresis and membrane transfer were carried out. A total of 50 mL of 5 % skimmed milk incubation solution was prepared, the membrane was rinsed with PBST, and incubated in skimmed milk for 1 h. Primary antibody was added and the membrane was incubated overnight at 4 °C. The membrane was then rinsed with PBST, the secondary antibody was added and the membrane was incubated at room temperature for 1 h. Take exposure photos.

### Statistical methods

The data were analyzed using, and the measurement data were expressed by x ± s. Use Shapiro Wilk test to evaluate the normality of continuous variables. Use analysis of variance (three group comparison) or t-test (two group comparison) to analyze data that conforms to a normal distribution. Use Bonferroni method to correct for multiple comparisons (p<0.05 with statistical significance, p<0.01 has statistical significance). Use a 95 % confidence interval. Post-hoc tests: Tukey’s HSD test was used after ANOVA and Dunn’s test after Kruskal−Wallis. Multiple comparisons correction: Bonferroni correction was used. All statistical analyses were conducted using GraphPad Prism version 9 (USA) software.

## Results

**Figure 1:**
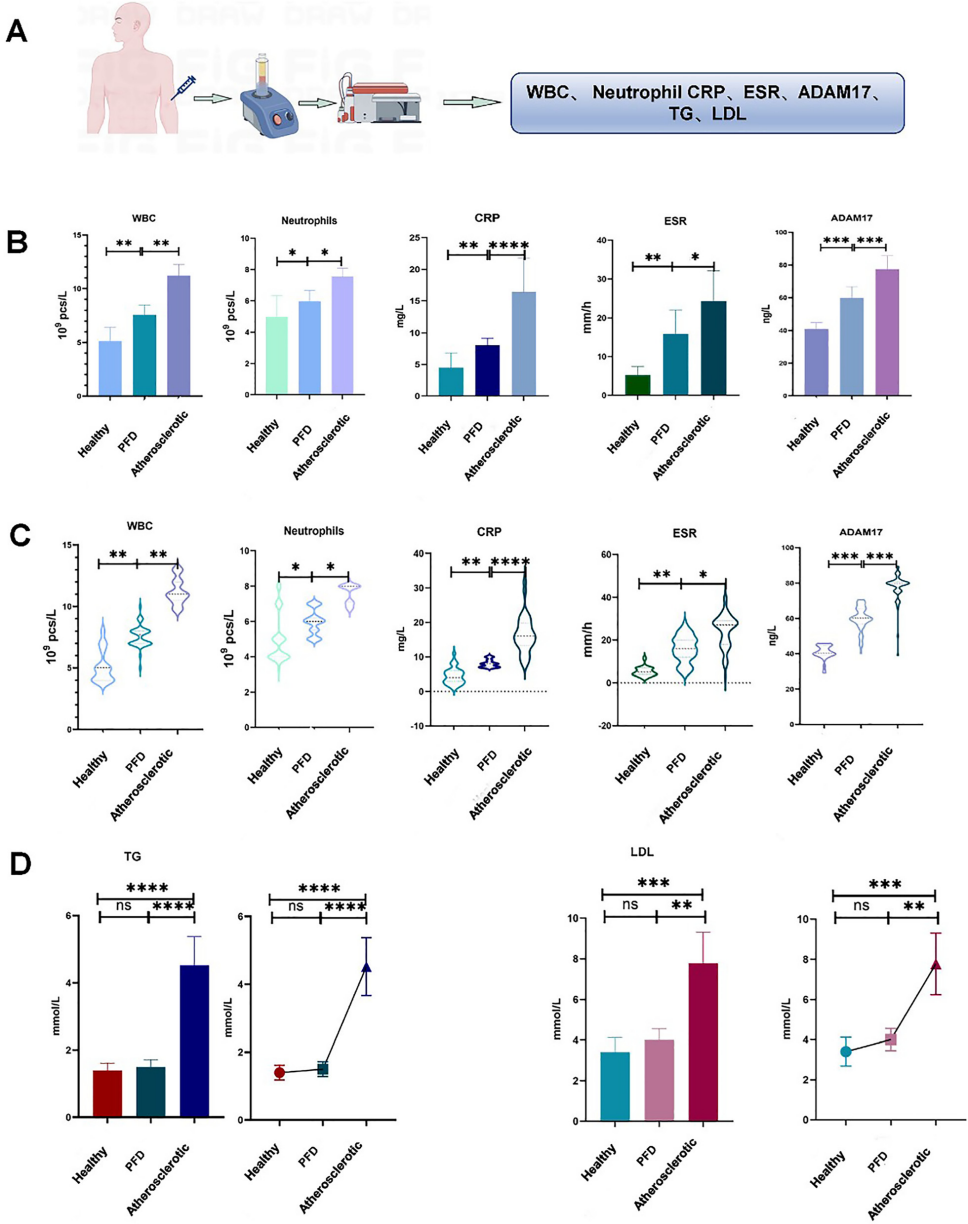
A is a schematic diagram of the experimental process; B is the expression of inflammation related factors, which is statistically significant in the health group and PFD group, PFD group and atherosclerosis group (p<0.001); C is a violin chart used to visualize the distribution and show significant statistical differences between the three groups (p<0.001); D refers to the difference in triglycerides and low-density lipoprotein (LDL) between healthy group, PFD group and atherosclerosis group. This shows that ADAM17 expression in healthy group, PFD group and atherosclerosis group has statistical significance.

*Expression of inflammatory factors in human serology is associated with AS, and ADAM17 is involved in atherogenesis*. Serological tests were carried out on leukocytes, neutrophils, C-reactive protein (CRP) and erythrocyte sedimentation rate (ESR) of different groups. The results showed that there were differences between the healthy group and the atherosclerotic (AS) group and the plaque-free dyslipidemia group. Furthermore, ADAM17 was found to be active between healthy and plaque free dyslipidemia groups were significantly different from AS groups, and both were significantly different from the AS group (p<0.001; Figure 1B and C). However, regarding TG and LDL indices, it was found that there was no difference between the healthy group and the plaque free dyslipidemia group, but there was a significant difference between the plaque free dyslipidemia group and the AS group (p<0.05; Figure 1D). The data suggest that ADAM17 is involved in the development of AS.

**Figure 2:**
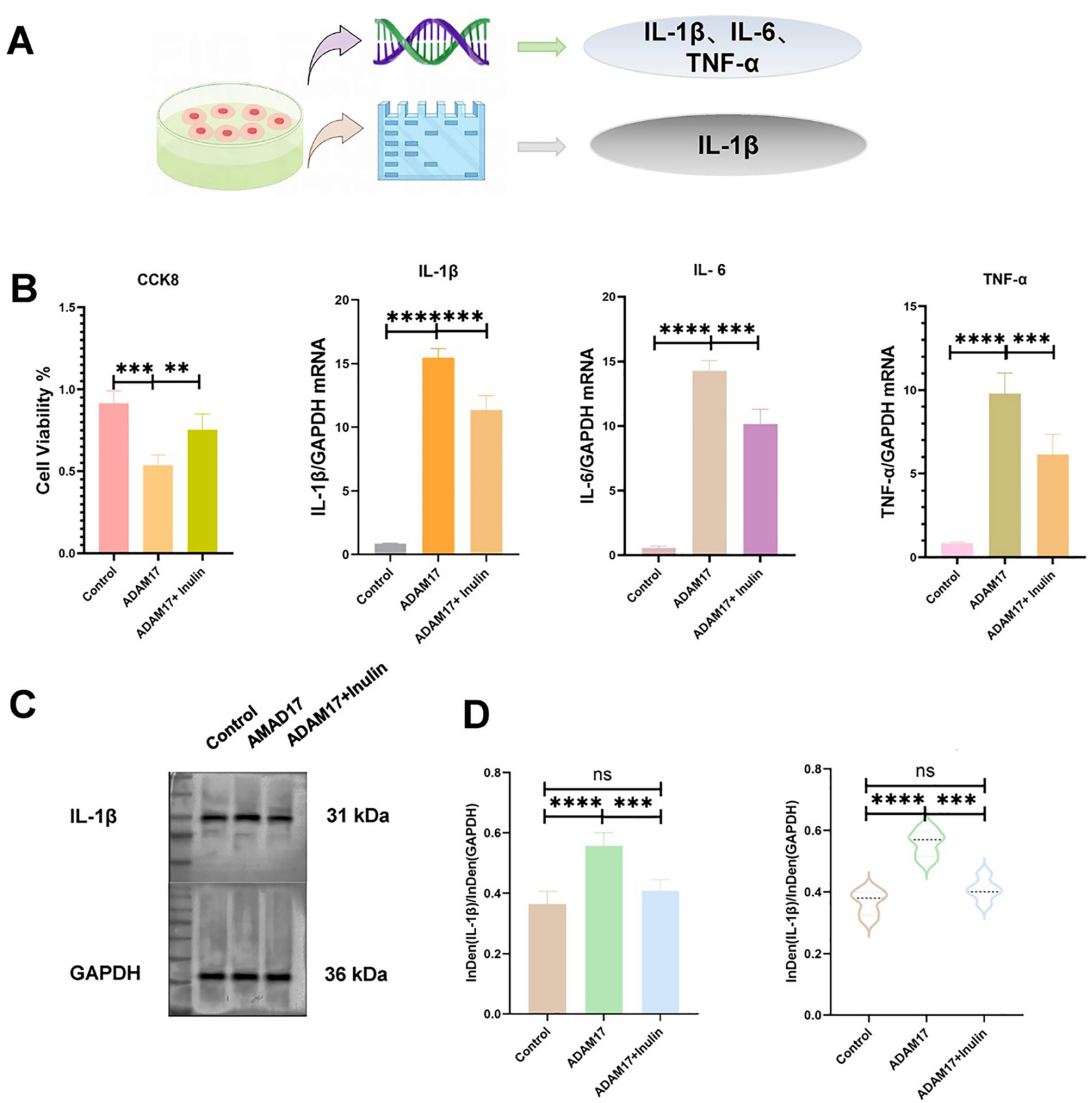
A is the cellular experimental procedure; B is the difference in the expression of inflammatory factors in the ADAM17 group and the ADAM17+Inulin group (p<0.001); C is the result of the Western blot experiment, and D is the statistical result of the Western blot experiment, and IL-1β was different between the two groups.

*Through RT-qPCR and western blotting experiments, the expression of inflammatory factors was found to be reduced in the ADAM17 + INU group*. ADAM17 and INU were added to different subsets of experiments in cell cultures, and the expression of IL-1β, IL-6 and TNF-α was found to be significantly different between the control and the ADAM17 groups. ADAM17 and ADAM17 + INU groups were different between the control and the ADAM17 + INU groups as examined by RT-qPCR (p<0.001; Figure 2B). were significantly different (p<0.001; Figure 2B). In addition, significant differences (p<0.001) in the protein expression of IL-1β were observed between the control and the ADAM17 groups, ADAM17 and ADAM17 + INU, and between the control and the ADAM17/ + INU groups (Figure 2C and D).

**Figure 3:**
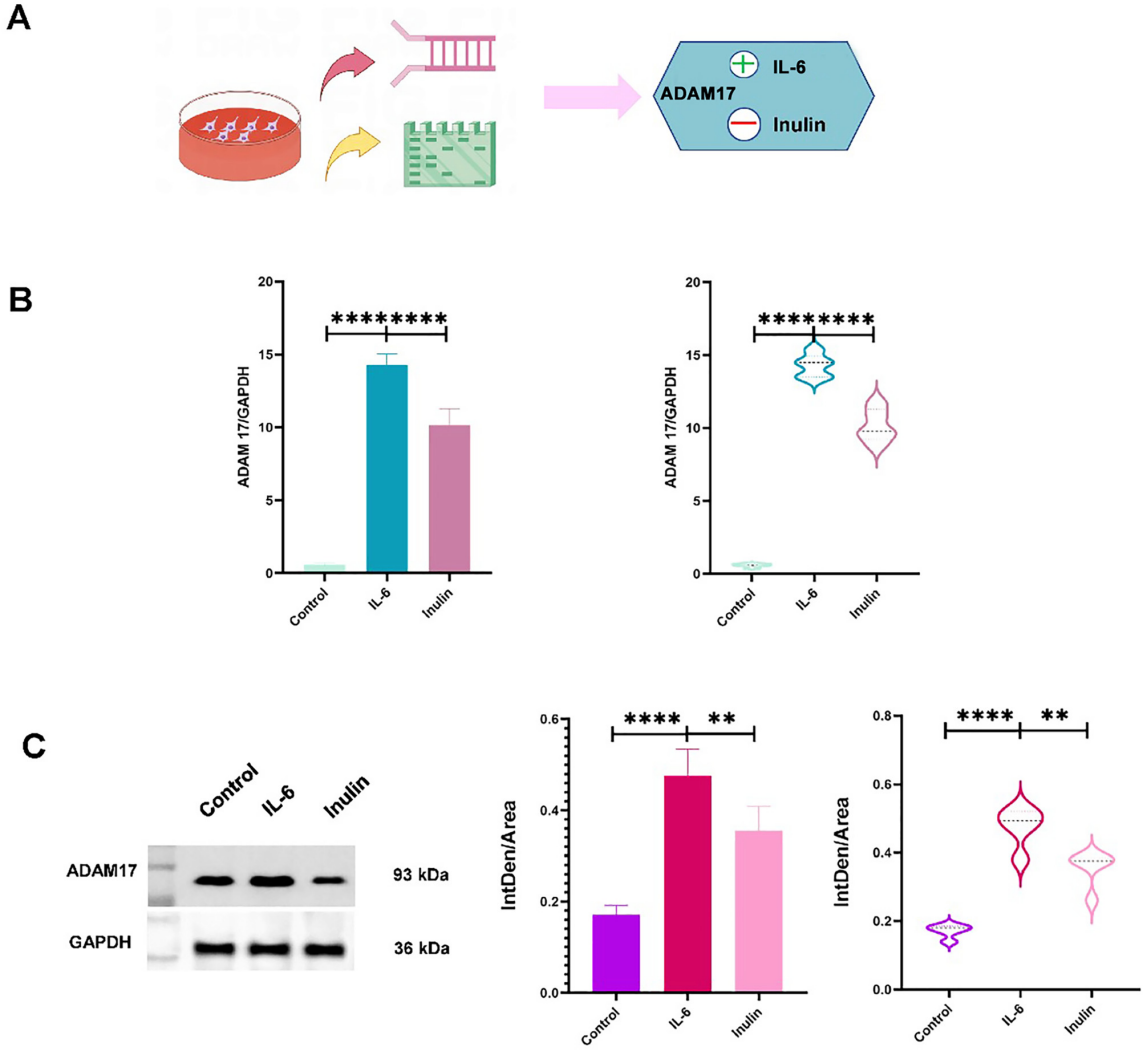
A is the experimental procedure; B is the RT-PCR result indicating that there is a difference in ADAM17 expression between IL-6 and Inulin groups (p<0.001); C is the result of Western blot experiment; D is the statistical result of Western blot experiment that there is a difference in ADAM17 expression between IL-6 and Inulin groups (p<0.001).

*A decrease in ADAM17 expression in the INU group was detected by RT-qPCR and western blotting*. Significant differences in ADAM17 protein expression between the control and the IL-6 groups, IL-6 and INU groups, and the control and the INU groups were confirmed by RT-qPCR (p<0.001; Figure 3B). In addition, it was also confirmed by western blotting that ADAM17 protein expression was decreased in the INU group compared with that in the IL-6 group, and the difference was significant (p<0.001; Figure 3C and D). IL-6 could promote ADAM17 expression and INU could inhibit it, and both showed an opposite effect.

**Figure 4:**
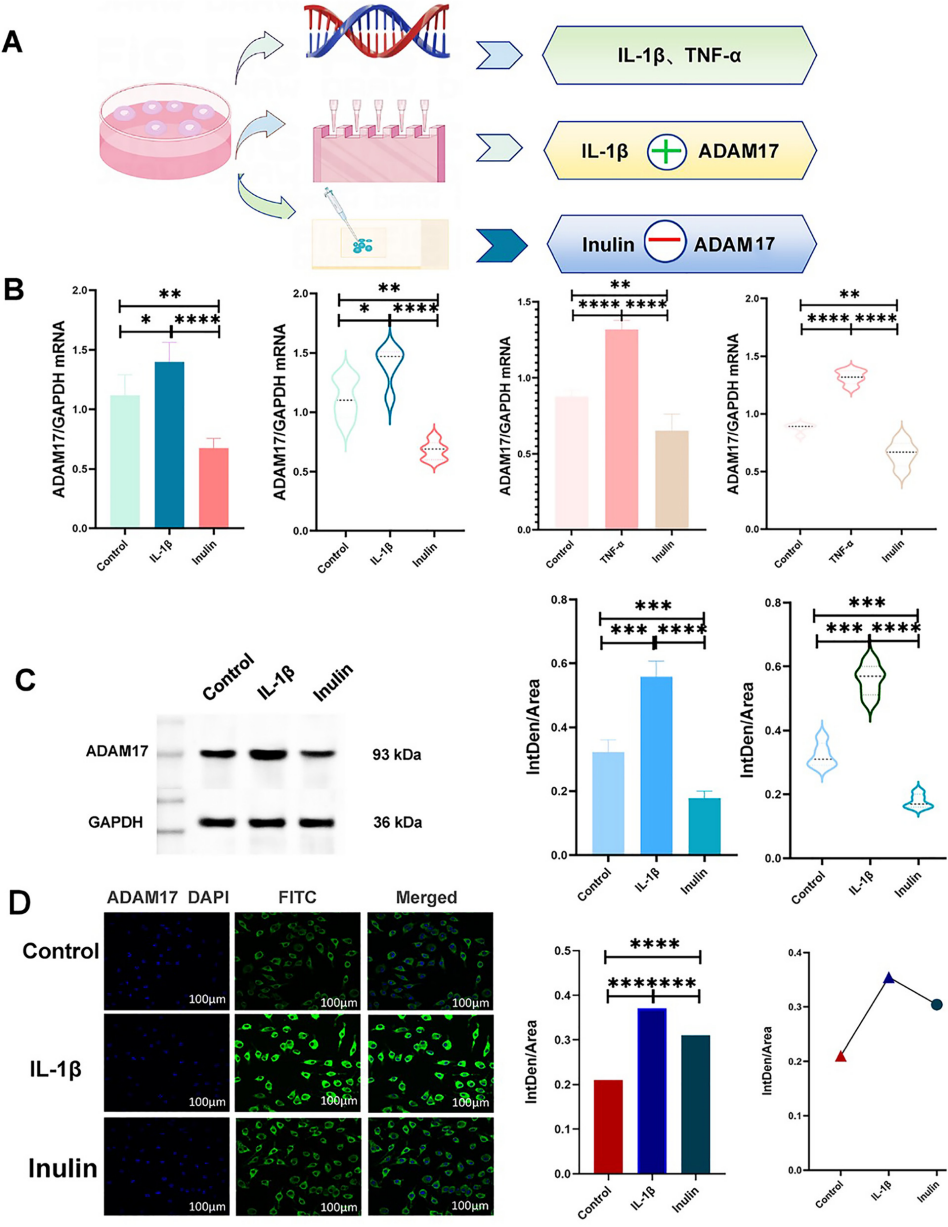
A is the experimental procedure; B is the RT-PCR results indicating that there is a difference in ADAM17 expression between IL-1β group and TNF-α and Inulin group (p<0.001); C is the results of Western blot experiments indicating that there is a difference in ADAM17 expression between IL-1β and Inulin groups (p<0.001); D is the results of immunofluorescence experiments that there was a difference in ADAM17 expression between the IL-1β group and the Inulin group.

*Inhibition of ADAM17 expression by INU was confirmed by RT-qPCR, western blotting and immunofluorescence experiments*. Significant differences in ADAM17 protein expression were observed between the control and the IL-1β groups, IL-1β and INU groups, and the control and the INU groups were confirmed by RT-qPCR (p<0.001; Figure 4B). Furthermore, a decrease in ADAM17 protein expression was also confirmed in the INU group compared with that in the IL-1β group by western blotting, and the difference was significant (p<0.001; Figure 4C). Immunofluorescence experiments revealed that ADAM17 protein expression was strongest in the IL-1β group and weakest in the control group. There was a significant difference in ADAM17 protein expression between the three groups (p<0.001; Figure 4D). IL-1β and TNF-α could promote ADAM17 expression and INU could inhibit ADAM17 expression. The two showed opposite effects to INU.

## Discussion

**Figure 5:**
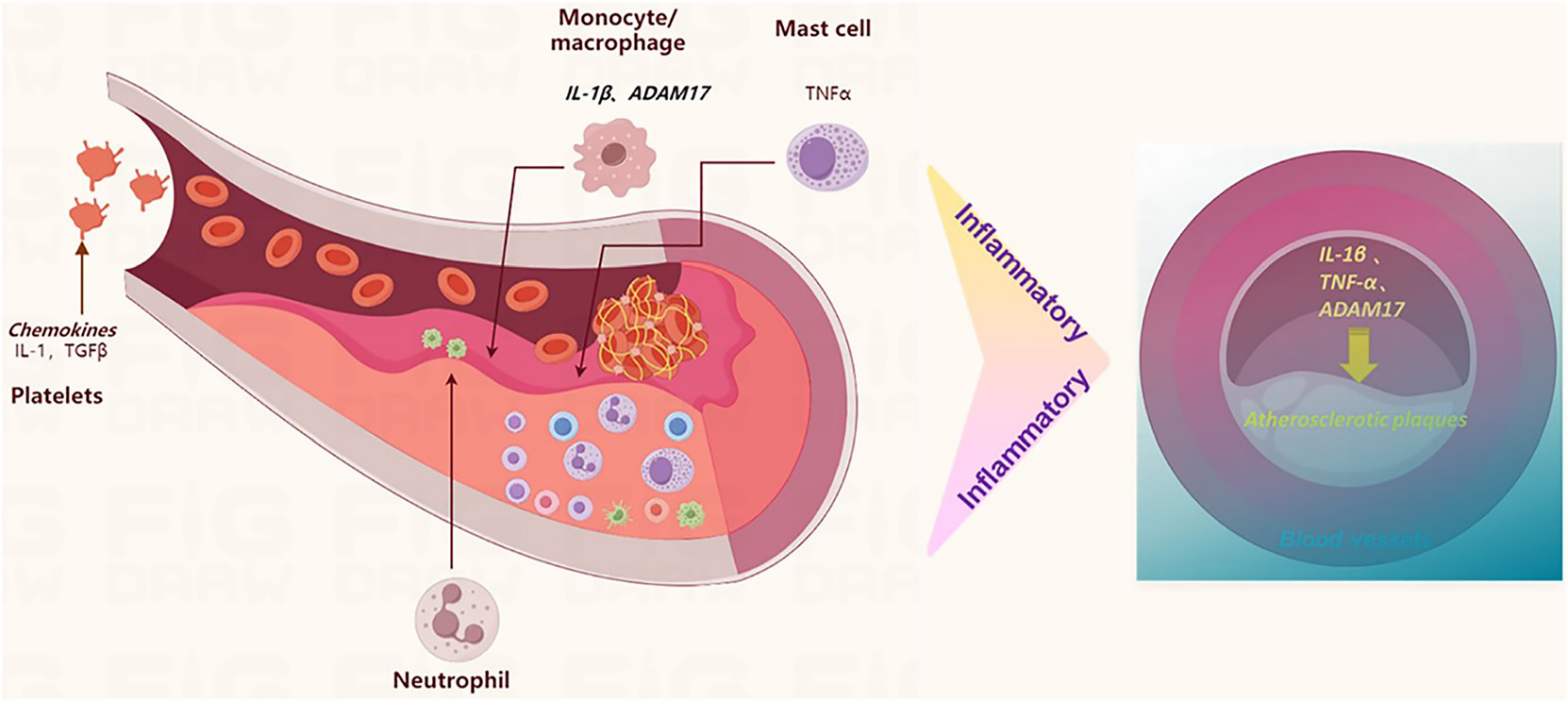
A diagram of the mechanism by which inflammation affects atherosclerosis in arterial vessels. Inflammatory factors lead to atherosclerosis of vascular inner wall (authors’ own drawing).

AS is a chronic inflammatory disease of the artery wall characterized by the formation of plaques containing lipids, connective tissue and immune cells in the intima of medium and large arteries. In the last three decades, a reduction in cardiovascular mortality has been achieved through LDL cholesterol-lowering therapies and therapies that target other traditional risk factors of cardiovascular disease [26]. Furthermore, Zhu et al. [27] found that compromised immune state and metabolic changes including high levels of inflammatory cytokines, disruption of lipid and mineral ion homeostasis, and accumulation of uremic toxins, during chronic kidney disease worsen the stability of AS plaques and promote vascular calcification. Furthermore, it is also a lipid-related inflammatory disease of the arterial mucosa, in which the balance of proinflammatory and inflammatory catabolic mechanisms determines the final clinical outcome [28]. It is also a disease characterized by mild chronic inflammation [29]. In conclusion, immunodeficiency directly contributes to increased vascular inflammation and worsens AS. It has also been found that patients with chronic inflammation, especially some patients with chronic intestinal inflammation [30] or chronic dental inflammation [31], are more susceptible to AS disease, which has been confirmed by our human studies. In addition, once inflammatory factors such as leukocytes, neutrophils, IL-1β and TNF-α are increased in the blood vessels, and then multiple factors such as high blood lipids and LDLs are present, AS changes in the lining of blood vessels occur. Blood vessels rapidly appear (Figure 5). In addition, this change was detected by studying ADAM17 and also it was hypothesized that inhibition of ADAM17 increases autoimmunity and thus slows down the AS effect.

The role of inflammation in AS was recognized decades ago, and existing treatments provide partial benefits through non-specific anti-inflammatory effects. In addition, the interaction of immune factors within the immune system also alleviates vascular inflammation [32]. Unlike other immune factors or cytokines, IL-1β, synthesized by monocytes, macrophages and dendritic cells [33], is associated with acute and chronic inflammation [34]. Inflammation of the vascular endothelium leads to endothelial dysfunction, thereby causing various pathological conditions, and inflammatory mediators in the vascular system precisely contribute to endothelial inflammation [35]. In addition, the results of the canakinumab anti-inflammatory thrombosis outcome study showed that IL-1β plays an important role in AS and that blocking IL-1β can treat AS diseases [36]. However, in the present study, the important immunomodulatory role of IL-1β in AS and the underlying mechanisms are discussed to show the changes in IL-1β observed by examining ADAM17 and INU using cell experiments. Finally, it was found that IL-1β expression was highest in the ADAM17 group, followed by the addition of the INU group. It was revealed that IL-1β, IL-6 and TNF-α can promote ADAM17 expression and INU can inhibit ADAM17 expression. It also suggested that the three molecules exhibited opposite effects to INU. It was also hypothesized that INU might slow the AS effect by inhibiting ADAM17 expression. According to similar studies, ADAM17 is a membrane protease that can trigger various signaling pathways and is closely linked to the occurrence of inflammation and other signals [37]. Chang et al. found that inulin enhances the anti-inflammatory effect of mouse intestinal apolipoprotein E4D [38]. Kawasaki et al. found that ADAM17 can alter vascular pathology in mice and humans by regulating TGF β signaling [39]. Therefore, inhibiting inflammation can reduce the changes in the vascular endothelium and thus improve AS.

In recent years, the number of relevant studies investigating the connection between IL-6 and arteriosclerosis has been on the increase. It is well known that aging promotes the development and progression of AS through various mechanisms. Furthermore, IL-6 signaling in bone marrow adipocytes increases with age, and studies have found that IL-6 is associated with age-related AS [40]. A recent study found that circulating IL-6 predicted the severity, susceptibility and progression of carotid plaques [41]. Further studies have found that the aging process is associated with neuroinflammation, characterized by the activation of immune cells in the central nervous system in response to injury, infection and other pathological stimuli [42], but persistent inflammatory stimuli increase the sub-risk of reduced immune function [43]. In some cases, this immune response becomes chronic and leads to the development of various neurological disorders.

In summary, AS is associated with inflammatory factors, whereas INU promotes cardiovascular and metabolic health [44]. However, the mechanisms involved in these protective processes are not well understood. According to Yu et al. [45], INU can markedly alleviate arthritis in CIA mice by regulating Th17/Treg balance and influencing immune inflammation. Jangid et al. [46] revealed that INU strengthens immunity by activating immune cells and promoting cytokine secretion, and also strengthens immunity by modifying cells. These studies have shown that INU can boost host immunity. Chronic low-grade inflammation is a known contributor to gastroesophageal reflux disease (GERD) pathogenesis [47]. By promoting a healthier gut environment and reducing inflammatory triggers, inulin supplementation might indirectly alleviate GERD symptoms or modify its progression. GERD and atherosclerosis may share underlying inflammatory pathways [48]. Therefore, the anti-inflammatory effects of inulin, mediated through gut microbiota modulation, could represent a common protective mechanism against both conditions. We propose that dietary interventions with prebiotics like inulin might not only benefit gastrointestinal health but also contribute to cardiovascular risk reduction by mitigating shared inflammatory pathways.

In recent years, numerous studies have indicated that inulin (INU) can improve triglycerides (TGs) and apolipoprotein E (ApoE) levels to mitigate atherosclerosis (AS) [49], 50]. However, no research has yet explored the direct influence of INU on AS progression or its specific effects on inflammatory factors. Accordingly, this study investigated IL-1β, TNF-α, and IL-6, demonstrating that inulin may attenuate ADAM17-mediated inflammation and thereby modulate vascular AS. It has also been proposed that inhibiting these inflammatory factors could either compromise host immunity or serve as a therapeutic target in AS. Our research lacks cellular functional assays and *in vivo* animal validation, which is a limitation of our study, but it can lay the foundation for our future work research.

By reducing ADAM17 expression, inulin may indirectly enhance immune function and promote overall health. The current findings provide strategic guidance and a novel reference for leveraging anti-inflammatory approaches to improve the prognosis of patients with AS.
